# Combination of serum histidine and plasma tryptophan as a potential biomarker to detect clear cell renal cell carcinoma

**DOI:** 10.1186/s12967-017-1178-8

**Published:** 2017-04-06

**Authors:** Hyung-Ok Lee, Robert G. Uzzo, Debra Kister, Warren D. Kruger

**Affiliations:** 1 Cancer Biology Program, Fox Chase Cancer Center, 333 Cottman Avenue, Philadelphia, PA 19111 USA; 2 Department of Surgical Oncology, Fox Chase Cancer Center, 333 Cottman Avenue, Philadelphia, PA 19111 USA

**Keywords:** Kidney cancer, Biomarker, Serum, Plasma, Amino acids

## Abstract

**Background:**

In previous work, we showed that serum-free amino acid (SFAA) profiles were different between kidney cancer patients and age and sex matched controls. The goals of the current study are to: (1) confirm our initial observation on an independent sample set; (2) examine if there were similar differences in plasma-free amino acids (PFAA); and (3) determine if removal of tumors changed SFAA and PFAA profiles.

**Methods:**

SFAA and PFAA profiles were measured in 484 samples taken from 124 healthy controls and 56 clear cell renal cell carcinoma (ccRCC) patients both before and after resection of renal tumors.

**Results:**

SFAA and PFAA profiles taken from identical blood samples were remarkably different, with the mean individual amino acid concentrations being 40% less in plasma compared to serum. Both SFAA and PFAA profiles differed significantly between ccRCC patients and controls, but the individual amino acids that differed the most, and the direction of the changes, were quite different between the two blood components. Removal of the tumor had almost no effect on either the SFAA or PFAA profiles. A logistic regression model using serum histidine and plasma tryptophan correctly classified 85.5% of control and 84.7% of case samples.

**Conclusions:**

Our findings show that that tumor mass is not directly linked to alterations in blood amino acid levels, and that a combination of serum histidine and plasma tryptophan may be useful as a biomarker to detect ccRCC.

**Electronic supplementary material:**

The online version of this article (doi:10.1186/s12967-017-1178-8) contains supplementary material, which is available to authorized users.

## Background

In the United States, there are over 50,000 cases of renal cell carcinoma diagnosed annually resulting in more than 13,000 deaths [1]. It is the eighth leading cause of cancer deaths in men and the fourteenth in woman. Prognosis is very much dependent on the stage at which the disease is caught. Small tumors confined to the kidney (T1) have 5-year survival rates as high as 90%, while advanced tumors that have metastasized outside the kidney (T4) have rates <20% [2]. Unfortunately, most individuals with locally confined disease have no obvious symptoms and, therefore, the disease is often detected at an advanced stage. Increasing the percentage of individuals diagnosed with early stage disease would lead to a significant reduction in mortality from this disease. The development of an inexpensive blood based screening test to detect renal cell carcinoma would be extremely useful in achieving this goal.

Our lab has previously published a study of free amino acids in preoperative serum (SFAA) taken from 189 renal cell carcinoma patients (65% clear cell) and 104 controls [3]. We found significant differences in 15 of the 26 amino acids assayed. A logistic regression model using 8 amino acids was able to distinguish controls from cases with an ROC of 0.81. This same model also had predictive value in terms of overall survival and tumor recurrence in patients with renal cell carcinoma.

Here, we asked the question if the alterations in SFAA profiles observed in renal cell carcinoma patients were directly due to the tumor mass itself, as renal cancer is known to have profound effects on cellular metabolism [4]. To answer this, we examined SFAA profiles in 56 new ccRCC patients that had blood samples taken both before and after exenterative surgery. In addition, we examined PFAA profiles in the same patients and compared them to controls. Our findings indicate that ccRCC patients have significant alterations in both PFAA and SFAA profiles, but removal of the tumor mass did not appreciably affect these differences.

## Methods

### Patients and samples

Serum and plasma were obtained from ccRCC patients (n = 56) and controls (n = 124) from the Fox Chase Cancer Center Biosample Repository (Additional file 1: Table S1). For serum, blood was collected in yellow-capped serum separating tubes, while plasma blood was collected in acid citrate dextrose vacutainers. Serum and plasma were collected at the same time. After processing, all samples were stored at −70 °C. All ccRCC cases were FCCC patients with histopathologically confirmed clear cell renal cancers who underwent exenterative surgery. Pre-surgical blood was collected within 30 days of surgery (56 samples of serum and plasma each). Post-surgical samples were collected after surgery with a median time of 238 days (28–1218 days). For some patients, multiple post-surgical samples were taken (62 samples of serum and plasma each). Control samples were matched with patient samples based on sex and age. These samples came from a variety of sources including FCCC employees, individuals undergoing routine cancer screening, or spouses of patients. None of the patient samples used in this study overlap with our previous study [3].

### Amino acid analysis

Serum amino acid levels for each sample were quantified using a Biochrom 30 amino acid analyzer as previously described [5]. Each sample was assayed once, as inter-day assay repeatability was previously established by processing 27 different samples on two different days resulting in an average CV for all of the amino acids of 6.7% (range 3.5–14.2%).

### Data analysis

For univariate analysis, unpaired two-sided t tests were used with p < 0.05 being deemed significant with no correction for multiple testing. An unpaired test was deemed appropriate, as there was not one-to-one matching between cases and controls. One-way ANOVA, in combination with Tukey’s LSD test, was used to determine differences between multiple groups. To identify the most predictive amino acids in either serum or plasma, forward step-wise regression was performed using all 25 amino acids as variables. Because we observed minimal differences in ccRCC samples taken before and after surgery, we combined these groups. At each step, the most predictive variable was included based on the Wald score. The final model contained only those variables with Wald scores with p < 0.05. All statistics were performed using Statistica 13 software (Dell Computer).

## Results

### Differences between PFAA and SFAA in controls

**Fig. 1:**
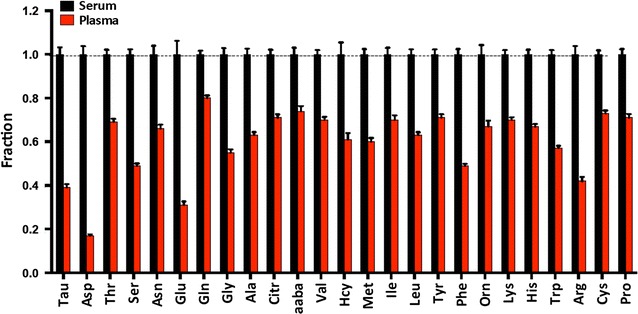
Serum (n = 124) versus plasma (n = 124) relative amino acid concentrations in controls. All serum amino acids have been normalized to 1, while plasma levels for each amino acid are shown as a fraction of serum. *Error bars* show SEM

We initially examined SFAA and PFAA profiles in 124 non-cancer controls (Fig. 1; Additional file 1: Table S2). Unexpectedly, there were large differences in the concentration of free amino acids in serum and plasma. Free amino acids were significantly reduced in plasma compared to serum, with a mean reduction of 40%, with all amino acids showing highly significant p values. However, the magnitude of the differences varied quite widely, ranging from a 20% reduction (glutamine) to a whopping 83% reduction (aspartate). The mean co-efficient of variation (CV) was quite similar between serum and plasma (32 vs. 31%, p = ns), suggesting that processing variability was not a factor. In ccRCC samples, the difference between serum and plasma was less, with a mean reduction of 23% (range 10–74%). The difference in behavior between control and RCC patients with regards to serum and plasma will be explored in more detail later.

### SFAA and PFAA in ccRCC patients versus controls

**Fig. 2:**
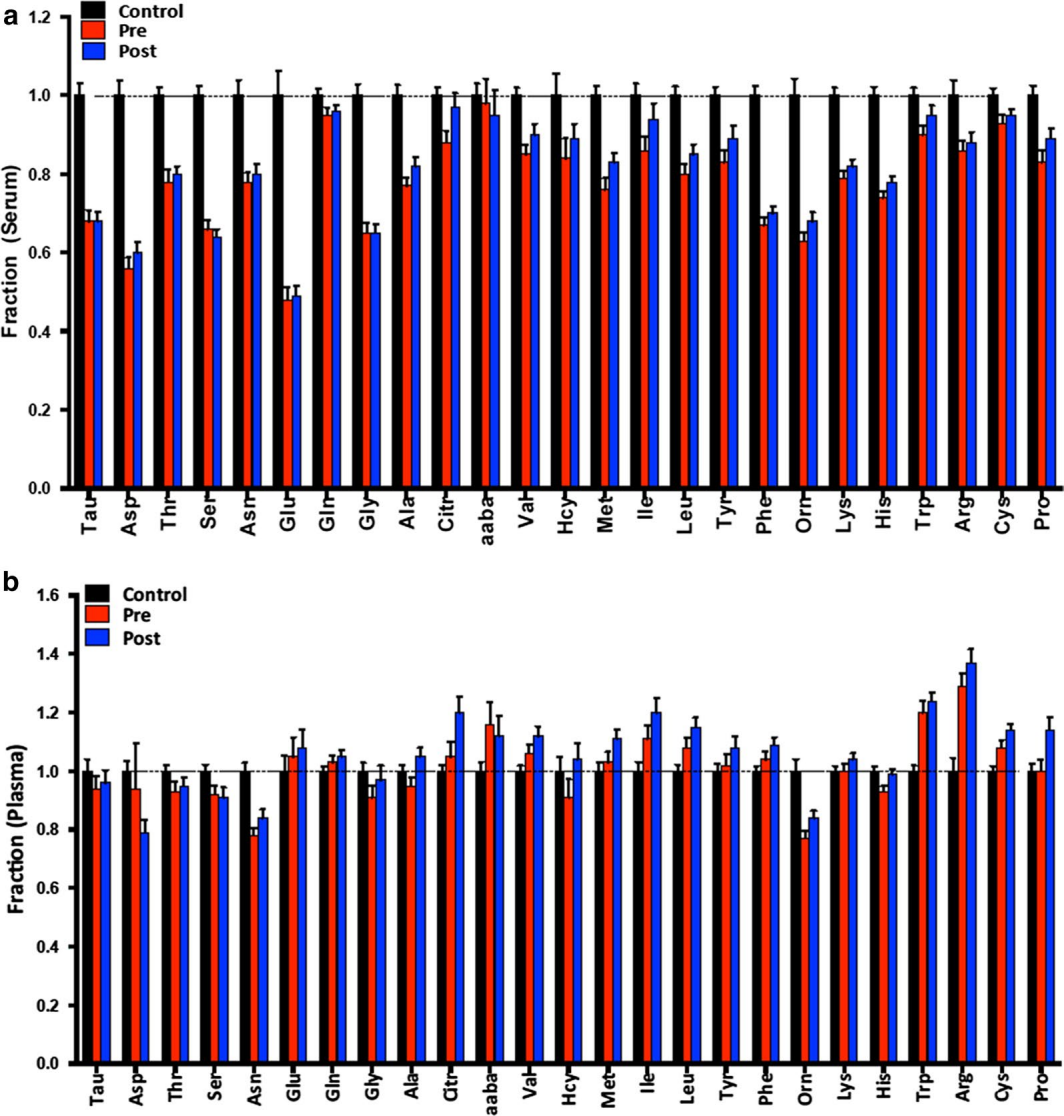
Comparison of controls, pre-surgery ccRCC and post-surgery ccRCC samples. **a** Relative serum amino acid concentrations compared to control. **b** Relative plasma amino acid concentrations compared to controls. *Error bars* show SEM

We compared serum amino acid concentrations in controls, ccRCC patients before surgery, and ccRCC patients after surgery (Fig. 2a; Additional file 1: Table S3). We found that the p value for the one-way ANOVA comparing these three groups was significant (p < 0.05) for 22 of the 25 amino acids measured and was highly significant (p < 0.0001) for 17 of these. Post-hoc testing revealed that, in almost all cases, the significance was driven by the difference between the controls and ccRCC samples (both pre- and post-surgery). In every case where the ANOVA was significant, ccRCC samples (combined pre- and post) had reduced concentrations of SFAA compared to controls with a mean reduction of 22%. The amino acids with the greatest percent decrease were glutamate, aspartate, serine, glycine, and ornithine. There were no statistically significant differences between ccRCC samples taken pre verse post surgery, although in 23 out of 25 amino acids, the mean did rise slightly, i.e., moved toward the control values.

Results in plasma were somewhat different (Fig. 2b; Additional file 1: Table S4). The one-way ANOVA was significant (p < 0.05) for 14 plasma amino acids, and five were highly significant (p < 0.0001). Unlike serum, there were at least three distinct patterns. Some amino acids (asparagine, ornithine, serine, and histidine) behaved similarly to serum, i.e. lower in both pre and post-surgical patients compared to controls. However, others (tryptophan, arginine, cysteine, citrulline, leucine, isoleucine, valine, phenylalanine, and methionine) were elevated in patients compared to controls. Finally, three amino acids (proline, alanine, and homocysteine) were slightly elevated only in post-surgical patient samples.

We also compared SFAA and PFAA profiles from individuals with early stage (I and II) versus late stage (III and IV) ccRCC. In both serum and plasma, we did not observe any statistically significant differences in any of the 25 amino acids (Additional file 1: Table S5). These findings indicate that alterations in PFAA and SFAA profiles are similar in both early and late stage ccRCC.

### Interaction effect between plasma, serum, and cancer

Our univariate analysis showed significant differences between cancer patients and controls for a number of amino acids in both serum and plasma. However, many amino acids showed very different behaviors with regards to both the blood component and the ccRCC status. For example, arginine was *decreased* by 13% in the serum of ccRCC patients, but was *increased* by 33% in plasma. To explore this further, we performed 2-way ANOVA analysis for each amino acid, examining the contribution of blood component (serum or plasma), ccRCC status, and the interaction effect between them (Additional file 1: Table S6). The total amount of variance for each amino acid that could be explained by these three factors ranged from a low of 6.8% (α-aminobutyric acid) to a high of 65.9% (aspartate), with the mean being 35%. Twenty-three of the 25 amino acids examined had statistically significant interaction terms, with the size of the interaction effect often being larger than the effect of isolated ccRCC status. These findings indicate that for most amino acids, ccRCC status affects plasma and serum differently.

### Modeling

We next performed forward-stepwise logistic regression to identify amino acids that could best separate ccRCC cases from controls for both serum and plasma. In serum, a six amino acid model including histidine, asparagine, serine, arginine, glycine, and α-aminobutyric acid classified 89.8% of cases and 84.7% of controls accurately, giving a receiver operator area under the curve (ROC) of 0.928 (Additional file 2: Fig. S1a). In plasma, the predictive amino acids were tryptophan, asparagine, cysteine, arginine, aspartate, and serine (Additional file 2: Fig. S1b). These correctly classified 84.7% of cases and 83.1% of controls with an ROC of 0.917.

**Fig. 3:**
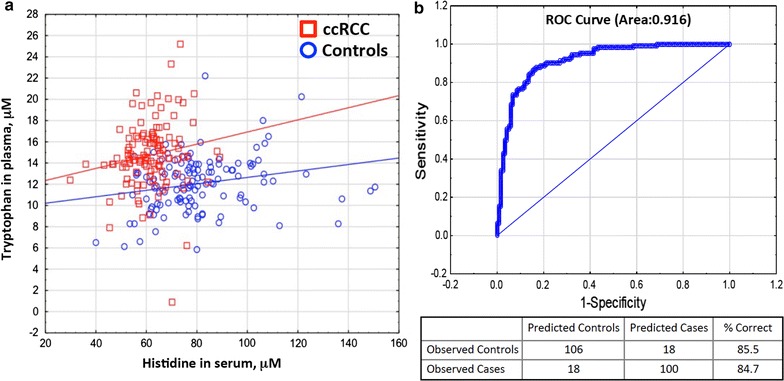
Scatter plot of serum histidine and plasma tryptophan for ccRCC samples and controls (**a**). Regression line for each group is shown (**b**)

Based on the Wald statistic, the most significant amino acids were histidine in serum and tryptophan in plasma. A scatterplot of cases and controls shows impressive separation of cases and controls using these two variables (Fig. 3a). A logistic regression model using just these two measures correctly predicts 85.5% of the controls and 84.7% of the cases with an ROC of 0.916 (Fig. 3b).

## Discussion

Here, we examined PFAA and SFAA in patients with ccRCC, before and after surgery, and compared them to healthy controls. There were four important findings from this study that will be discussed below.

First, we found that amino acid concentrations differ remarkably between plasma and serum. In our control patients, plasma had on average a 40% reduction in free-amino acid concentrations compared to serum. This was a surprise to us, as serum is assumed to be equivalent to plasma without the clotting factors. In searching the literature, we were able to find only one other study [6] that examined the differences between serum and plasma using the same type of tubes (acid citrate) as the ones used here. This group also found that plasma had reduced free amino acid pools compared to serum, although our mean reduction was somewhat larger (27 vs. 40%). Why are free-amino acid levels so different between serum and plasma? One possibility is that the increased level of amino acids found in serum is due to the addition of the contents of platelets that are activated as part of the coagulation process. Platelets that become activated release the contents of various granules into the bloodstream [7]. Lysosomes, which play a role in amino acid sensing and storage, are among the granules that are released [8]. If this idea is correct, our data suggests that the amount of amino acids present in these granules is not insignificant.

The second important finding is that there are large differences in both the PFAA and SFAA profiles between ccRCC patients and controls. Our finding that the serum of ccRCC patients tend to have reduced levels of amino acids is consistent with our earlier study [3]. In the current study, we found that 20 amino acids were decreased and zero increased in a statistically significant manner, but in our previous study, only 13 amino acids were decreased and two were increased. A possible explanation for the more robust results described here may be because the earlier study combined both ccRCC patients with other types of renal cancer including papillary, chromophobe, and mixed sub-types, suggesting a more heterogeneous population. Unique to this study was our findings in plasma, which was not analyzed in the earlier study. Far fewer amino acids were altered in plasma, and the two showing the greatest difference (tryptophan and arginine) were actually elevated in the ccRCC patients. Logistic regression modeling indicates that serum did a slightly better job at classifying cases and controls than plasma. Impressively, a two-factor model using plasma tryptophan and serum histidine classified cases and controls nearly as well as a six component plasma model and a six component serum model. One important weakness of the current study is that we have only focused on ccRCC and do not know if other types of cancer may show similar alterations. Also, it is possible that alterations in amino acids may be related to behavioral or dietary differences between patients and controls [9, 10].

A third important finding was that the differences between plasma and serum amino acid concentrations were differentially affected by ccRCC status. In controls, we saw 40% mean reduction in free amino acid concentrations in plasma compared to serum, but in ccRCC cases, there was only a 23% difference. Two-way ANOVA showed that there was a statistically significant interaction effect between blood component and ccRCC status for 23 of the 25 amino acids. This finding suggests that ccRCC has unique effects on both serum and plasma, and that these effects are different in the different blood components. The underlying reason behind this is not clear, but since the difference between plasma and serum concerns clotting factors, it is possible that ccRCC patients may have some alterations in haemostatic factors. Interestingly, high plasma fibrinogen levels predicted poorer outcome in a European cohort of non-metastatic renal cell carcinoma patients [11].

Finally, our most unexpected finding was that removal of the ccRCC tumor mass had minimal effect on the PFAA and SFAA profiles. In our previous paper, we had speculated that the generally lower levels of serum amino acids might be a reflection of the increased usage of amino acids by the tumor for biosynthetic processes [3]. However, our data here indicates that the tumor is not directly responsible, since our after surgery samples still show similar PFAA and SFAA profiles even though they have been debulked of their tumors. We can think of two possible explanations for this finding. First, it is possible that altered PFAA and SFAA profiles represent a sort of “ccRCC susceptibility profile,” i.e., people in the population with these profiles are enriched in the sub-set that later develops ccRCC. In theory, this could be explored by examining PFAA and SFAA profiles in a prospective study design. Second, it is possible that the altered profiles represent some sort of secondary response to the cancer, which persists even after the cancer is removed. For example, one might imagine that some sort of immune response or inflammatory response to the cancer persists even when the bulk of the offending antigen has been removed.

## Conclusions

In summary, our data shows that while both SFAA and PFAA profiles are altered in ccRCC patients compared to healthy controls, this alteration does not seem to be directly linked to tumor mass. Future studies will need to focus on whether differences in amino acid profiles are specific to ccRCC, or whether they occur in other types of cancers as well. Also, longitudinal studies studies will need to be performed to determine if a combination of serum histidine and plasma tryptophan might be a useful as a biomarker for early detection of ccRCC or other cancers.

## Additional files

**Additional file 1: Table S1.** Sample characteristics. **Table S2.** Serum and plasma amino acid concentrations in controls. **Table S3.** Serum amino acid concentrations. **Table S4.** Plasma amino acid concentrations. **Table S5.** Comparison SFAA and PFAA profiles with tumor stage. **Table S6.** Interaction effect between serum, plasma, and cancer.

**Additional file 2: Figure S1.** **a, b**. ROC curves for logistic regression models from SFAA and PFAA.

## Abbreviations

SFAA: serum free amino acids
PFAA: plasma free amino acids
ccRCC: clear cell renal carcinoma
