# Supplementary material for: Combination of serum histidine and plasma tryptophan as a potential biomarker to detect clear cell renal cell carcinoma
Source: J Transl Med. 2017 Apr 6;15:72. doi: 10.1186/s12967-017-1178-8 (PMC5383954; doi:10.1186/s12967-017-1178-8)
Supplement: Supplementary file 1 — Additional file 1: Table S1. Sample characteristics. Table S2. Serum and plasma amino acid concentrations in controls. Table S3. Serum amino acid concentrations. Table S4. Plasma amino acid concentrations. Table S5. Comparison SFAA and PFAA profiles with tumor stage. Table S6. Interaction effect between serum, plasma, and cancer. [file 12967_2017_1178_MOESM1_ESM.docx]

**Additional file 1: Table S1. Sample characteristics**

|  |  |  |  |
| --- | --- | --- | --- |
|  | Patients | Controls | t-test (p) |
| Total patients | 56 | 124 |  |
|  |  |  |  |
| Age |  |  |  |
| Median | 62 | 59 | 0.57 |
| Range | 24 - 78 | 25 - 81 |  |
|  |  |  |  |
| Sex (%) |  |  |  |
| Male | 40 (71) | 83 (67) | 0.33 |
| Female | 16 (29) | 40 (32) |  |
|  |  |  |  |
| Race (%) |  |  |  |
| Known (n) | 55 | 71 |  |
| White | 46 (84) | 65 (92) | 0.517 |
| Black | 8 (14) | 6 (8) |  |
| Asian | 1 (2) | 0 (0) |  |
|  |  |  |  |
| BMI (%) |  |  |  |
| Known (n) | 55 | 55 |  |
| Median | 30.2 | 28 | 0.064 |
| Range | 16.3 - 48.2 | 18.3 - 55.2 |  |
|  |  |  |  |
| Stage (%) |  |  |  |
| I | 2 (4) |  |  |
| II | 25 (45) |  |  |
| III | 21 (38) |  |  |
| IV | 8 (14) |  |  |

**Additional file 1: Table S2. Serum and plasma amino acid concentrations in controls**

|  | Control Serum | |  | Control Plasma | |  |  |
| --- | --- | --- | --- | --- | --- | --- | --- |
| Amino acids | Mean (μM) | SD |  | Mean (μM) | SD |  | t-test (p) |
| Taurine | 147.3 | 50.4 |  | 58 | 26.8 |  | 2.20E-49 |
| Aspartate | 29.6 | 12.6 |  | 5.1 | 1.9 |  | 8.34E-46 |
| Threonine | 129.6 | 30.2 |  | 89.2 | 21.3 |  | 1.36E-57 |
| Serine | 132.8 | 34.6 |  | 64.9 | 15.6 |  | 1.65E-55 |
| Asparagine | 78 | 34.1 |  | 51.2 | 16.1 |  | 2.05E-19 |
| Glutamate | 147.4 | 102.1 |  | 45.3 | 27.8 |  | 1.46E-26 |
| Glutamine | 458.9 | 84.3 |  | 368.5 | 59.8 |  | 2.98E-35 |
| Glycine | 312 | 98 |  | 172 | 53.7 |  | 1.74E-50 |
| Alanine | 466.7 | 133.8 |  | 296.2 | 70.4 |  | 2.54E-45 |
| Citrulline | 34.6 | 8.1 |  | 24.5 | 5.9 |  | 2.70E-66 |
| α-aminobutyric acid | 18.2 | 6.1 |  | 13.5 | 4.6 |  | 2.14E-52 |
| Valine | 234.7 | 48.6 |  | 163.9 | 33.8 |  | 8.26E-62 |
| Homocysteine | 12.5 | 7.6 |  | 7.7 | 4.2 |  | 3.52E-27 |
| Methionine | 24.2 | 6.5 |  | 14.5 | 4.5 |  | 4.32E-53 |
| Isoleucine | 59.1 | 19.6 |  | 41.5 | 13.8 |  | 1.85E-43 |
| Leucine | 140.3 | 35.3 |  | 89 | 21.9 |  | 7.99E-55 |
| Tyrosine | 64 | 15.9 |  | 45.6 | 11.5 |  | 8.45E-57 |
| Phenylalanine | 84.9 | 22.7 |  | 41.3 | 8.5 |  | 6.41E-50 |
| Ornithine | 118.1 | 56.5 |  | 78.6 | 35 |  | 1.45E-33 |
| Lysine | 192.1 | 41.1 |  | 134.1 | 25.2 |  | 4.04E-56 |
| Histidine | 80.8 | 19.2 |  | 53.8 | 10.1 |  | 2.09E-51 |
| Tryptophan | 21.3 | 4.4 |  | 12.1 | 2.6 |  | 1.86E-71 |
| Arginine | 85.7 | 35.8 |  | 35.9 | 18 |  | 4.39E-45 |
| Cysteine | 320.5 | 65.2 |  | 232.8 | 47.1 |  | 1.33E-63 |
| Proline | 230.5 | 62.1 |  | 163.6 | 44.6 |  | 2.97E-53 |

**Additional file 1: Table S3. Serum amino acid concentrations^1^**

|  | Pre-surgery |  | Post-surgery |  | Controls |  |  |
| --- | --- | --- | --- | --- | --- | --- | --- |
|  | (n=56) |  | (n=62) |  | (n=124) |  |  |
| Amino acids | Mean (μM) |  | Mean (μM) |  | Mean (μM) |  | Anova ( p) |
| Taurine | 100.5 ^a^ |  | 100.7 ^a^ |  | 147.3 ^b^ |  | < 0.0001 |
| Aspartate | 16.6 ^a^ |  | 17.7 ^a^ |  | 29.6 ^b^ |  | < 0.0001 |
| Threonine | 101.5 ^a^ |  | 104.2 ^a^ |  | 129.6 ^b^ |  | < 0.0001 |
| Serine | 88.0 ^a^ |  | 84.9 ^a^ |  | 132.8 ^b^ |  | < 0.0001 |
| Asparagine | 60.9 ^a^ |  | 62.6 ^a^ |  | 78.0 ^b^ |  | < 0.0001 |
| Glutamate | 70.3 ^a^ |  | 72.2 ^a^ |  | 147.4 ^b^ |  | < 0.0001 |
| Glutamine | 437.7 ^a^ |  | 439.4 ^a^ |  | 458.9 ^a^ |  | 0.1085 |
| Glycine | 203.2 ^a^ |  | 203.8 ^a^ |  | 312.0 ^b^ |  | < 0.0001 |
| Alanine | 357.6 ^a^ |  | 382.2 ^a^ |  | 466.7 ^b^ |  | < 0.0001 |
| Citrulline | 30.5 ^a^ |  | 33.7 ^a,b^ |  | 34.6 ^b^ |  | 0.0107 |
| α-aminobutyric acid | 17.7 ^a^ |  | 17.3 ^a^ |  | 18.2 ^a^ |  | 0.7346 |
| Valine | 199.5 ^a^ |  | 211.9 ^a^ |  | 234.7 ^b^ |  | < 0.0001 |
| Homocysteine | 10.5 ^a^ |  | 11.2 ^a^ |  | 12.5 ^a^ |  | 0.1056 |
| Methionine | 18.4 ^a^ |  | 20.2 ^a^ |  | 24.2 ^b^ |  | < 0.0001 |
| Isoleucine | 50.9 ^a^ |  | 55.4 ^a,b^ |  | 59.1 ^b^ |  | 0.0209 |
| Leucine | 112.6 ^a^ |  | 119.4 ^a^ |  | 140.3 ^b^ |  | < 0.0001 |
| Tyrosine | 52.9 ^a^ |  | 56.7 ^a^ |  | 64.0 ^b^ |  | < 0.0001 |
| Phenylalanine | 56.5 ^a^ |  | 59.7 ^a^ |  | 84.9 ^b^ |  | < 0.0001 |
| Ornithine | 74.0 ^a^ |  | 80.4 ^a^ |  | 118.1 ^b^ |  | < 0.0001 |
| Lysine | 151.0 ^a^ |  | 158.3 ^a^ |  | 192.1 ^b^ |  | < 0.0001 |
| Histidine | 59.8 ^a^ |  | 63.3 ^a^ |  | 80.8 ^b^ |  | < 0.0001 |
| Tryptophan | 19.1 ^a^ |  | 20.2 ^a,b^ |  | 21.3 ^b^ |  | 0.004 |
| Arginine | 73.3 ^a^ |  | 75.7 ^a,b^ |  | 85.7 ^b^ |  | 0.0095 |
| Cysteine | 298.7 ^a^ |  | 303.4 ^a,b^ |  | 320.5 ^b^ |  | 0.0248 |
| Proline | 192.4 ^a^ |  | 205.4 ^a^ |  | 230.5 ^b^ |  | < 0.0001 |

^1^ All concentrations are micromolar and the mean is shown. Entries with different superscript letters in same row are statistically different (P<0.05) as determined by post-hoc testing.

**Additional file 1: Table S4. Plasma amino acid concentrations^1^**

|  | Pre-surgery |  | Post-surgery |  | Controls |  |  |
| --- | --- | --- | --- | --- | --- | --- | --- |
|  | (n=56) |  | (n=62) |  | (n=124) |  |  |
| Amino acids | Mean (μM) |  | Mean (μM) |  | Mean (μM) |  | Anova ( p) |
| Taurine | 54.4 ^a^ |  | 55.8 ^a^ |  | 58.0 ^a^ |  | 0.6015 |
| Aspartate | 4.8 ^a^ |  | 4.0 ^a^ |  | 5.1 ^a^ |  | 0.0984 |
| Threonine | 82.8 ^a^ |  | 84.4 ^a^ |  | 89.2 ^a^ |  | 0.1107 |
| Serine | 59.5 ^a^ |  | 59.3 ^a^ |  | 64.9 ^a^ |  | 0.0266 |
| Asparagine | 40.0 ^a^ |  | 42.8 ^a^ |  | 51.2 ^b^ |  | < 0.0001 |
| Glutamate | 47.5 ^a^ |  | 48.8 ^a^ |  | 45.3 ^a^ |  | 0.6439 |
| Glutamine | 381.0 ^a^ |  | 387.1 ^a^ |  | 368.5 ^a^ |  | 0.1045 |
| Glycine | 156.4 ^a^ |  | 167.6 ^a^ |  | 172.0 ^a^ |  | 0.2318 |
| Alanine | 280.9 ^a^ |  | 311.2 ^a^ |  | 296.2 ^b^ |  | 0.052 |
| Citrulline | 25.6 ^a^ |  | 29.5 ^b^ |  | 24.5 ^a^ |  | 0.0004 |
| α-aminobutyric acid | 15.6 ^a^ |  | 15.1 ^a^ |  | 13.5 ^a^ |  | 0.0634 |
| Valine | 173.4 ^a,b^ |  | 182.8 ^a^ |  | 163.9 ^b^ |  | 0.0047 |
| Homocysteine | 7.0 ^a^ |  | 8.0 ^a^ |  | 7.7 ^a^ |  | 0.3507 |
| Methionine | 15.0 ^a,b^ |  | 16.1 ^a^ |  | 14.5 ^b^ |  | 0.0464 |
| Isoleucine | 46.2 ^a,b^ |  | 49.7 ^a^ |  | 41.5 ^b^ |  | 0.0011 |
| Leucine | 96.5 ^a,b^ |  | 102.5 ^a^ |  | 89.0 ^b^ |  | 0.0005 |
| Tyrosine | 46.3 ^a^ |  | 49.1 ^a^ |  | 45.6 ^b^ |  | 0.1915 |
| Phenylalanine | 42.8 ^a,b^ |  | 45.2 ^a^ |  | 41.3 ^b^ |  | 0.0138 |
| Ornithine | 60.1 ^a^ |  | 66.2 ^a^ |  | 78.6 ^b^ |  | < 0.0001 |
| Lysine | 134.3 ^a^ |  | 139.4 ^a^ |  | 134.1 ^b^ |  | 0.3389 |
| Histidine | 50.0 ^a^ |  | 53.5 ^a,b^ |  | 53.8 ^b^ |  | 0.0279 |
| Tryptophan | 14.5 ^a^ |  | 14.9 ^a^ |  | 12.1 ^b^ |  | < 0.0001 |
| Arginine | 46.1 ^a^ |  | 49.3 ^a^ |  | 35.9 ^b^ |  | < 0.0001 |
| Cysteine | 251.2 ^a^ |  | 264.4 ^a^ |  | 232.8 ^b^ |  | < 0.0001 |
| Proline | 163.1 ^a^ |  | 186.1 ^b^ |  | 163.6 ^a^ |  | 0.0083 |

^1^ All concentrations are micromolar and the mean is shown. Entries with different superscript letters in same row are statistically different (P<0.05) as determined by post-hoc testing.

**Additional file 1: Table S5.**  **Comparison SFAA and PFAA profiles with tumor stage^1^**

|  |  | Serum |  |  |  | Plasma |  |
| --- | --- | --- | --- | --- | --- | --- | --- |
| Amino acids | LG | HG | t-test (p) |  | LG | HG | t-test (p) |
| Taurine | 98.9 | 102.5 | 0.504 |  | 55.1 | 55.2 | 0.988 |
| Aspartate | 17.3 | 17 | 0.821 |  | 4.8 | 4 | 0.327 |
| Threonine | 103.4 | 102.4 | 0.835 |  | 83.8 | 83.5 | 0.949 |
| Serine | 84 | 88.9 | 0.222 |  | 57.5 | 61.3 | 0.195 |
| Asparagine | 62.1 | 61.6 | 0.862 |  | 41.3 | 41.6 | 0.894 |
| Glutamate | 74.7 | 67.7 | 0.24 |  | 50.8 | 45.4 | 0.173 |
| Glutamine | 440.1 | 437.1 | 0.796 |  | 383.6 | 384.8 | 0.91 |
| Glycine | 201.6 | 205.5 | 0.717 |  | 156 | 168.8 | 0.244 |
| Alanine | 372.9 | 368 | 0.728 |  | 291.5 | 302.3 | 0.369 |
| Citrulline | 32.9 | 31.4 | 0.361 |  | 28.7 | 26.6 | 0.27 |
| α-aminobutyric acid | 17.3 | 17.8 | 0.773 |  | 14.9 | 15.7 | 0.575 |
| Valine | 206.7 | 205.3 | 0.876 |  | 177.7 | 179.1 | 0.856 |
| Homocysteine | 10.5 | 11.3 | 0.303 |  | 7.3 | 7.7 | 0.505 |
| Methionine | 19.6 | 19.1 | 0.595 |  | 15.4 | 15.7 | 0.721 |
| Isoleucine | 53.2 | 53.2 | 0.997 |  | 47.4 | 48.6 | 0.689 |
| Leucine | 116.5 | 115.9 | 0.91 |  | 99 | 100.2 | 0.779 |
| Tyrosine | 54.6 | 55.2 | 0.854 |  | 47.1 | 48.5 | 0.586 |
| Phenylalanine | 58.6 | 57.8 | 0.719 |  | 44.7 | 43.4 | 0.389 |
| Ornithine | 78.2 | 76.5 | 0.642 |  | 64.2 | 62.4 | 0.532 |
| Lysine | 156.8 | 152.8 | 0.421 |  | 136.2 | 137.8 | 0.711 |
| Histidine | 63 | 60.3 | 0.121 |  | 52.7 | 50.9 | 0.215 |
| Tryptophan | 19.4 | 19.9 | 0.435 |  | 14.4 | 15.1 | 0.21 |
| Arginine | 75.9 | 73.2 | 0.403 |  | 49.2 | 46.3 | 0.192 |
| Cysteine | 299.6 | 302.8 | 0.692 |  | 257.9 | 258.4 | 0.945 |
| Proline | 203.8 | 194.4 | 0.321 |  | 178.1 | 172.2 | 0.558 |

^1^LG=low grade, (stage I and II) ; HG=high grade (stage III and IV)

**Additional file 1: Table S6. Interaction effect between serum, plasma, and cancer**

| Amino acid | % Patient/Control Effect (p) | % Blood Component Effect (p) | % Interaction Effect (p) |
| --- | --- | --- | --- |
| Taurine | 6.1 (<0.0001) | 44.8 (<0.0001) | 4.8 (<0.0001) |
| Aspartate | 6.7 (<0.0001) | 54.0 (<0.0001) | 5.3 (<0.0001) |
| Threonine | 7.1 (<0.0001) | 24.2 (<0.0001) | 3.1 (<0.0001) |
| Serine | 12.2 (<0.0001) | 40.9 (<0.0001) | 7.6 (<0.0001) |
| Asparagine | 6.7 (<0.0001) | 22.3 (<0.0001) | 0.4 (ns) |
| Glutamate | 6.8 (<0.0001) | 19.9 (<0.0001) | 7.9 (<0.0001) |
| Glutamine | 0.0 (ns) | 22.3 (<0.0001) | 1.4 (0.0034) |
| Glycine | 10.5 (<0.0001) | 24.6 (<0.0001) | 7.3 (<0.0001) |
| Alanine | 4.4 (<0.0001) | 28.4 (<0.0001) | 4.5 (<0.0001) |
| Citrulline | 0.0 (ns) | 15.9 (<0.0001) | 2.3 (0.0002) |
| a-aminobutyric acid | 0.2 (ns) | 5.9 (<0.0001) | 0.8 (0.0461) |
| Valine | 0.5 (ns) | 23.5 (<0.0001) | 4.5 (<0.0001) |
| Homocysteine | 0.7 (ns) | 13.5 (<0.0001) | 0.4 (ns) |
| Methionine | 2.3 (<0.0001) | 28.7 (<0.0001) | 5.7 (<0.0001) |
| Isoleucine | 0.0 (ns) | 10.3 (<0.0001) | 3.0 (<0.0001) |
| Leucine | 1.0 (0.0076) | 25.4 (<0.0001) | 6.6 (< 0.0001) |
| Tyrosine | 1.2 (0.0085) | 15.9 (<0.0001) | 3.1 (<0.0001) |
| Phenylalanine | 7.1 (<0.0001) | 41.6 (<0.0001) | 10.9 (<0.0001) |
| Ornithine | 11.6 (<0.0001) | 10.6 (<0.0001) | 2.4 (0.0001) |
| Lysine | 5.2 (<0.0001) | 25.1 (<0.0001) | 7.1 (<0.0001) |
| Histidine | 9.7 (<0.0001) | 29.3 (<0.0001) | 6.5 (<0.0001) |
| Tryptophan | 0.2 (ns) | 47.1 (<0.0001) | 4.4 (<0.0001) |
| Arginine | 0.0 (ns) | 39.9 (<0.0001) | 3.6 (<0.0001) |
| Cysteine | 0.1 (ns) | 29.0 (<0.0001) | 3.4 (<0.0001) |
| Proline | 0.7 (0.0432) | 14.8 (<0.0001) | 3.3 (<0.0001) |
