# Supplementary material for: Combination of serum histidine and plasma tryptophan as a potential biomarker to detect clear cell renal cell carcinoma
Source: J Transl Med. 2017 Apr 6;15:72. doi: 10.1186/s12967-017-1178-8 (PMC5383954; doi:10.1186/s12967-017-1178-8)
Supplement: Supplementary file 2 — Additional file 2: Figure S1. a, b. ROC curves for logistic regression models from SFAA and PFAA. [file 12967_2017_1178_MOESM2_ESM.pdf]

Additional file 2: Fig. S1a

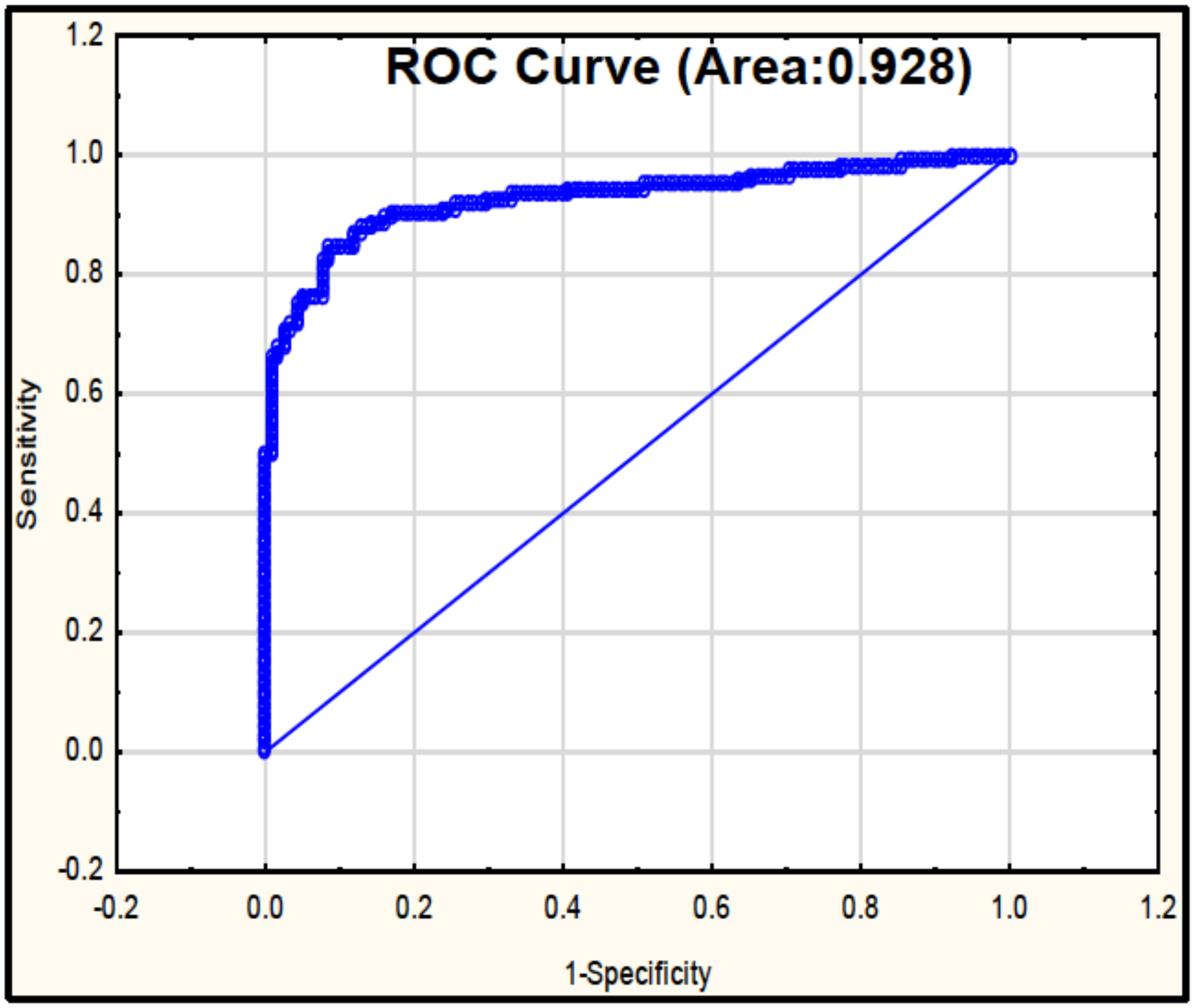

Effect	Wald scores	p
Intercept	47.8731	0.000000
Ser	10.4941	0.001198
His	22.5233	0.000002
aaba	4.8395	0.027815
Asn	11.7457	0.000610
Arg	6.5908	0.010251
Gly	4.8875	0.027052

	Predicted Controls	Predicted Cases	% Correct
Observed Controls	105	19	84.7
Observed Cases	12	106	89.8

Additional file 2: Fig. S1b

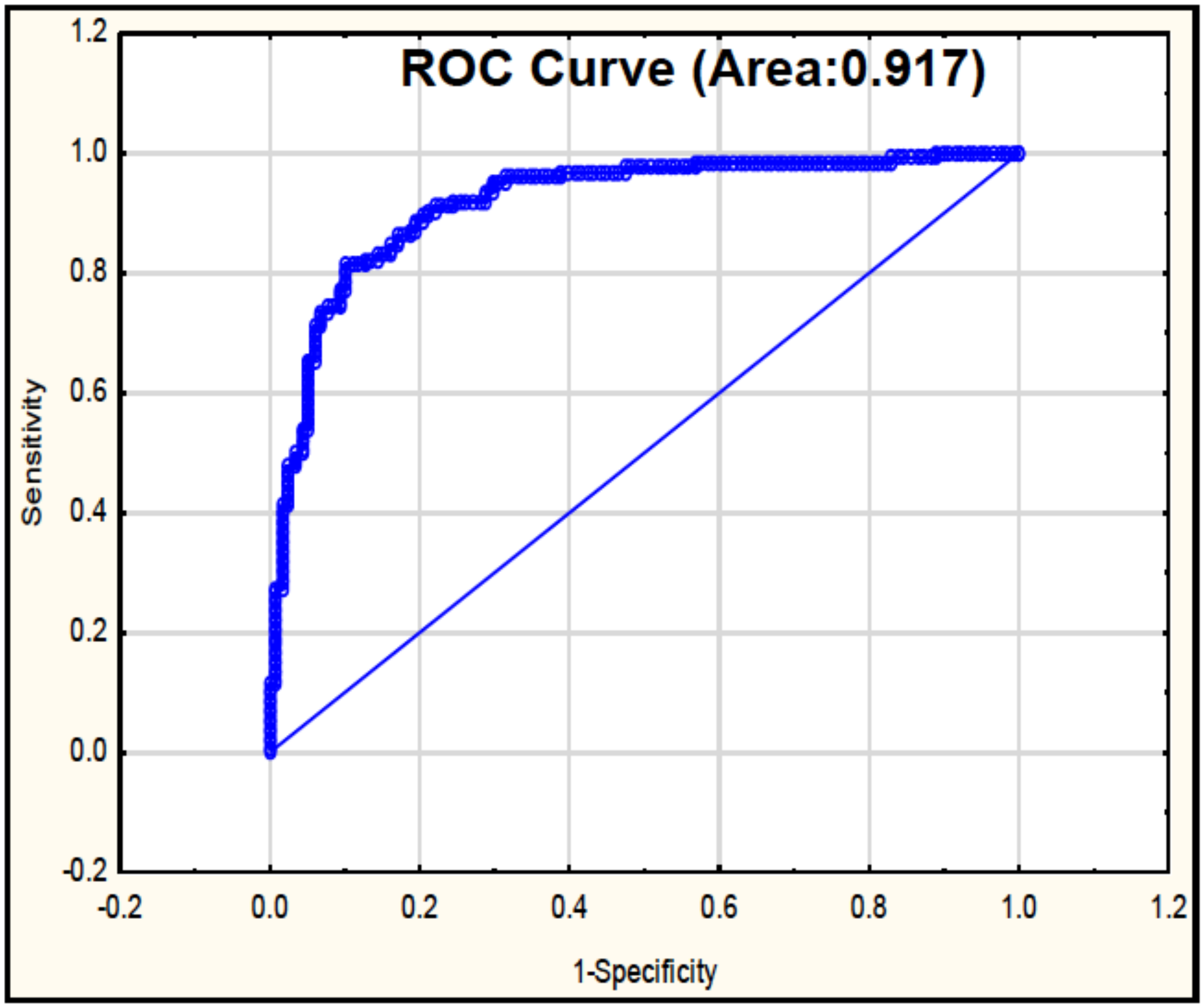

Effect	Wald scores	p
Intercept	30.4504	0.000000
Trp	36.4898	0.000000
Asn	37.1814	0.000000
Cys	21.2199	0.000004
Arg	14.6413	0.000130
Asp	9.4839	0.002073
Ser	5.1989	0.022601

	Predicted Controls	Predicted Cases	% Correct
Observed Controls	103	21	83.1
Observed Cases	18	100	84.7
